# TET (Ten-eleven translocation) family proteins: structure, biological functions and applications

**DOI:** 10.1038/s41392-023-01537-x

**Published:** 2023-08-11

**Authors:** Xinchao Zhang, Yue Zhang, Chaofu Wang, Xu Wang

**Affiliations:** 1 Department of Pathology, Ruijin Hospital and College of Basic Medical Sciences, Shanghai Jiao Tong University School of Medicine, Shanghai, 200025 China; 2 Key Laboratory of Cell Differentiation and Apoptosis of Chinese Ministry of Education, Shanghai Jiao Tong University School of Medicine, Shanghai, 200025 China

**Keywords:** Epigenetics, Epigenetics analysis

## Abstract

Ten-eleven translocation (TET) family proteins (TETs), specifically, TET1, TET2 and TET3, can modify DNA by oxidizing 5-methylcytosine (5mC) iteratively to yield 5-hydroxymethylcytosine (5hmC), 5-formylcytosine (5fC), and 5-carboxycytosine (5caC), and then two of these intermediates (5fC and 5caC) can be excised and return to unmethylated cytosines by thymine-DNA glycosylase (TDG)-mediated base excision repair. Because DNA methylation and demethylation play an important role in numerous biological processes, including zygote formation, embryogenesis, spatial learning and immune homeostasis, the regulation of TETs functions is complicated, and dysregulation of their functions is implicated in many diseases such as myeloid malignancies. In addition, recent studies have demonstrated that TET2 is able to catalyze the hydroxymethylation of RNA to perform post-transcriptional regulation. Notably, catalytic-independent functions of TETs in certain biological contexts have been identified, further highlighting their multifunctional roles. Interestingly, by reactivating the expression of selected target genes, accumulated evidences support the potential therapeutic use of TETs-based DNA methylation editing tools in disorders associated with epigenetic silencing. In this review, we summarize recent key findings in TETs functions, activity regulators at various levels, technological advances in the detection of 5hmC, the main TETs oxidative product, and TETs emerging applications in epigenetic editing. Furthermore, we discuss existing challenges and future directions in this field.

## Introduction

DNA methylation is one of the most common DNA modifications in mammals, and typically occurs at the CpG dinucleotide site where a methyl group is added to the fifth position of cytosine to generate 5-methylcytosine.^1–4^ This process is mediated by DNA methyltransferase (DNMTs). Among them, DNMT3a, DNMT3b, and DNMT3c establish de novo methylation by targeting unmethylated CpG sites, while DNMT1 predominantly serves as a maintenance methyltransferase during cell divisions.^5,6^ Although DNA methylation is generally stable, it can be removed by active demethylation associated with TET dioxygenases (DNA replication-independent) and passive demethylation (DNA replication-dependent). TET dioxygenases, specifically, TET1, TET2, and TET3 oxidize 5-methylcytosine (5mC) to 5-hydroxymethylcytosine (5hmC), 5-formylcytosine (5fC), and 5-carboxycytosine (5caC) in an Fe (II)/α-ketoglutarate-dependent manner.^7–9^ Notably, 5fC and 5caC can be excised by thymine-DNA glycosylase (TDG), and the modified site returns to the unmethylated status through base excision repair (BER).^10–14^ Therefore, these enzymes regulate active turnover of DNA methylation. Besides, UHRF1 recognizes 5mC:C dyads and recruits DNMT1 to hemi-methylated CpG sites to maintain DNA methylation.^15,16^ Disruption of this DNA methylation machinery dilutes 5mC during DNA replication. In addition, 5hmC reduces the affinity of UHRF1 towards 5hmC:C dyads and alters the specificity of DNMT1.^17–19^ Additionally, 5fC:C and 5caC:C dyads are capable of reducing the activity of DNMT1 in vitro.^20^ These observations suggest that all three oxidation products of TETs (5hmC, 5fC, and 5caC) are poor DNMT1 substrates and are involved in passive DNA demethylation.

*TET1* was the first identified member of *TET* family, acting as a fusion partner of *MLL* gene in acute myeloid leukemia patients bearing the t(10;11)(q22;q23) translocation, and *TET2* and *TET3* were subsequently identified based on their significant sequence homology to *TET1*.^21,22^ The biological function of TET family was unclear until two landmark discoveries by Kriaucionis et al. ^23^ and Tahiliani et al. ^7^. They found TET1 could covert 5mC to 5hmC, which was an Fe (II)/α-ketoglutarate-dependent enzyme by homology searching for JBP1, known as enzymes to oxidize methyl-thymine.^7,24,25^ Further findings revealed that TET2 and TET3 also could catalyze similar reactions.^8^ In addition to converting 5mC to 5hmC, TETs were capable of oxidating 5hmC to 5fC and further to 5caC.^9^

**Fig. 1:**
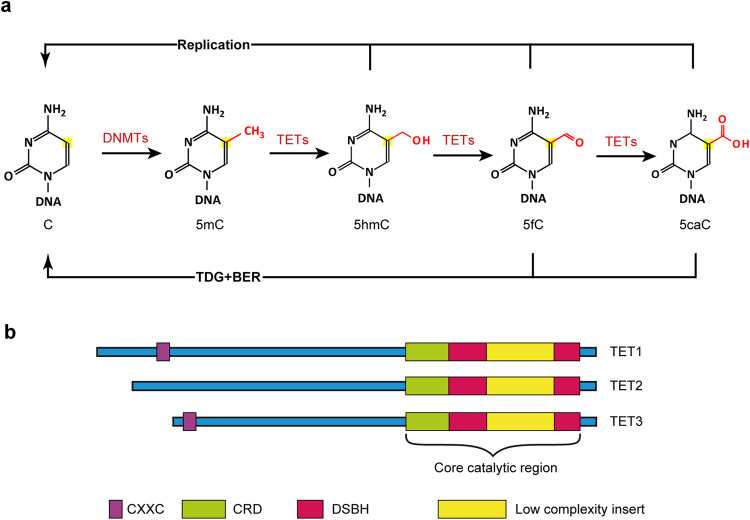
Function and structure of TET proteins. **a** The dynamic cycle of DNA methylation and demethylation. DNA methyltransferases (DNMTs) catalyzed the formation of 5-methylcytosine (5mC), which can be removed by TET-mediated oxidation, coupled with thymine-DNA glycosylase (TDG)-involved excision and base excision repair (BER). **b** Domain structure of TET proteins. All TET proteins possessed one core catalytic domain in C-terminal. A CXXC domain, located in the N-terminal of TET1 and TET3, but not in TET2, conferred DNA binding ability directly

The mechanism underlying TET-mediated demethylation of DNA was not clear until 2011, when two important papers identified that the oxidization products of 5mC, 5fC, and 5caC, could be excised by TDG,^10,11^ suggesting that TET-mediated oxidization was implicated in active DNA demethylation^26–28^ This was supported by the following study that biochemical reconstitution of TET-TDG-BER system could lead to DNA demethylation^29^(Fig. 1a).

Because DNA demethylation and the 5hmC mark involve in various biological reactions, TETs play a very important role in both physiological and pathological processes, which have been elucidated by many studies.^13,30–36^ For example, TET2 loss resulted in hypermutagenicity in haematopoietic progenitor cells, unveiling a key role of TET2 in safeguarding cells against genomic mutagenicity.^37^ Dysfunctions of TET2 in cancer are associated with *TET2* mutation and abnormal expression of TET2 regulators.^32,38–42^ Of note, since 2009, many studies have demonstrated that *TET2* mutations were frequently identified in multiple hematologic diseases.^43–49^ By genomic sequencing, one study revealed that *TET2* mutations were present in 14% (2 of 14) of patients with myelodysplastic syndrome (MDS), 37% (11 of 30) with myelodysplastic/myeloproliferative neoplasms (MDS/MPN), and 43% (6 of 14) with secondary acute myeloid leukemia (sAML) evolved from MDS/MPN. Among the patients harboring *TET2* mutations, MDS/MPN accounted for 58% (11 of 19), sAML evolved from MDS/MPN represented 32% (6 of 19), and MDS comprised 10% (2 of 19).^44^ However, *TET2* mutations were infrequent in patients with solid tumors,^50^ despite somatic mutations in *TET1*(8 of 74), *TET2*(5 of 74), and *TET3*(4 of 74) were identified in colon cancer.^51^ Other molecular mechanisms underlying the dysregulation of TETs functions in both blood and solid cancers were diverse and complex such as metabolic alterations.^52,53^ These are discussed in the part of TETs function regulators.

In the following sections, we discuss the structures, functions, and regulators of TETs and summarize the representative methods for 5hmC detection and epigenetic editing.

## TET family structure

The primary structure of TETs contains a carboxy-terminal catalytic domain, which is made up of a cysteine-rich domain (CRD), and two double-stranded β-helix (DSBH) regions separated by a large low-complexity insert.^7,25^ The DSBH domain possesses key residues, responsible for binding to its cofactors (α-ketoglutarate and Fe (II)), which are necessary to its catalytic function.^54^ Two zinc fingers combine the DSBH and CRD together to form the compact catalytic core.^54^ Although TET proteins are capable of oxidizing 5mC to 5hmC, 5fC and 5caC, structure analysis revealed that TET2 preferred 5mC substrate, rather than 5hmC and 5fC.^55^

TET1 and TET3 contain a CXXC domain, located in the amino-terminal region, which is implicated in binding to CpG dinucleotides,^56,57^ whereas TET2 loses its CXXC domain likely due to a chromosomal inversion (Fig. 1b). Consequently, this allows the ancestral TET2 CXXC domain to be a separate gene called *IDAX* (also named *CXXC4*). In the case of TET1 and TET3 with their respective CXXC domains, they can bind with DNA directly. In vitro binding assays revealed that TET1 slightly preferred substrates of unmethylated over that of methylated.^58^ Further studies showed that, similar to other proteins harboring the CXXC zinc finger domain, TET1 preferentially bound to CpG-enriched promoters of genes, which was certified by chromatin immunoprecipitation of TET1 coupled with DNA sequencing in mouse embryonic stem cells (mESCs).^59,60^ Similarly, TET3 CXXC-bound regions exhibited a significant enrichment of CpG and more than half of them were enriched in gene promoters.^57^ This study further revealed that the CXXC domain of TET3 was essential to its biological function through biochemical and structural analysis.^57^ In contrast, 5hmC regulated by TET2 is mainly located in gene bodies and exons rather than gene promoters.^61^ Of note, TET1/3 can be also recruited by their binding proteins for context-specific DNA regions.^62,63^ For example, the pluripotency factor NANOG interacted with TET1 and ChIP-seq analysis identified TET1-NANOG co-binding sites associated with NANOG target genes, suggesting that NANOG guided TET1 to specific sites of chromatin and some DNA-binding proteins were also important to TET1 functions.^62^

## TETs functions and binding partners

**Fig. 2:**
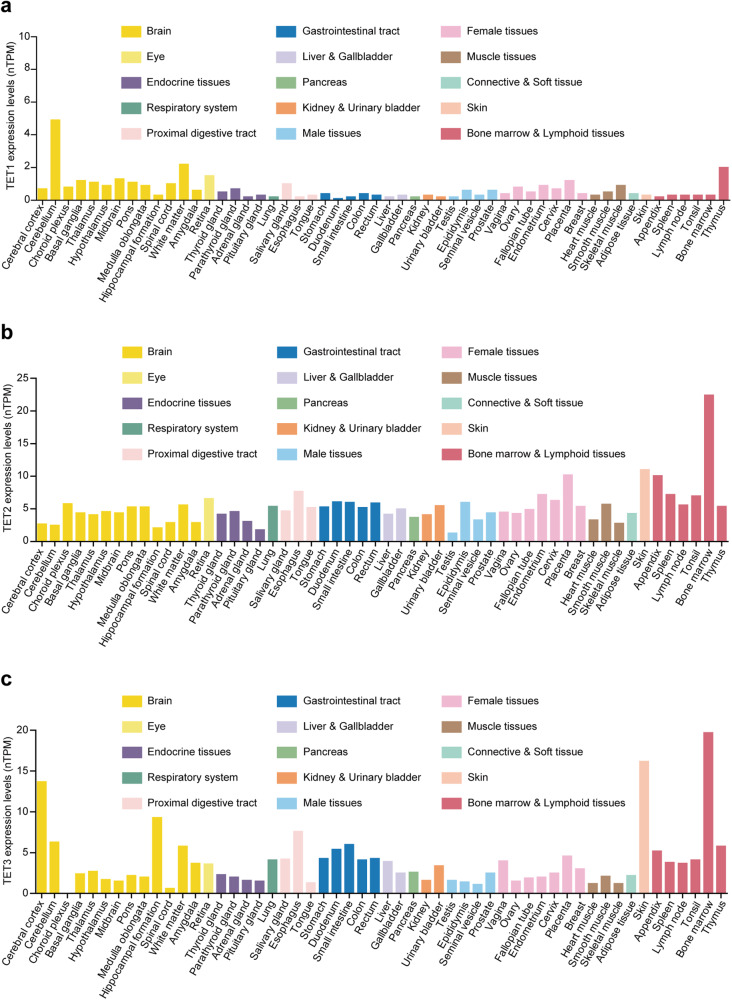
Expression levels of *TET1*(a), *TET2*(b), and *TET3*(c) in different tissues. The data were obtained from the proteinatlas.^67^ Modified from images available for TET1 (https://www.proteinatlas.org/ENSG00000138336-TET1/tissue), for TET2 (https://www.proteinatlas.org/ENSG00000168769-TET2/tissue), and for TET3(https://www.proteinatlas.org/ENSG00000187605-TET3/tissue). nTPM: consensus normalized expression

**Fig. 3:**
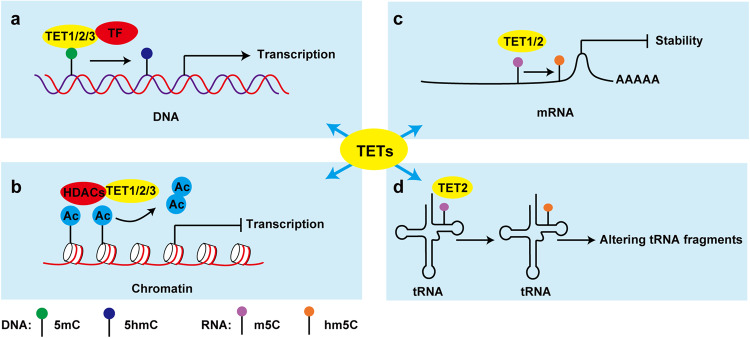
TETs working models. **a** A particular transcription factor (TF) recruited TETs to specific DNA areas of promoters to increase the downstream gene expression in a dioxygenase activity-dependent manner.^100,120^ **b** TETs binding with other epigenetic regulatory enzymes, such as HDAC, together regulated the particular gene expression independent of TETs enzyme activity.^183,187^ **c**, **d** TET1/2 oxidated mRNA (**c**)^191,194^ and TET2 oxidated tRNA (**d**)^196^ to exhibit regulatory functions in RNA levels

The primary functions of TETs are able to oxidate 5mC, and the products are subsequently involved in DNA demethylation.^64–66^ Besides, TET genes expression in different tissues are analyzed using the proteinatlas database^67^(https://www.proteinatlas.org/) in Fig. 2, which may suggest unique and various functions of TETs in tissues. Evidences also support 5hmC as an epigenetic mark, not only a demethylation intermediate.^68^ In addition, the non-catalytic activities of TETs are discovered.^69^ In this section, we discuss the classical and non-classical functions of TETs (Fig. 3). As TETs binding partners appear to be their main regulators, we also summarize their information here (Tables 1–3).

**Table 1:** Different types of TET1 binding proteins and their functions

Type	Name[reference]	Effects	TET1 catalytic dependent; substrates
Transcription factors	PPARγ^335^	Recruit TET1 to specific DNA sites; Enhance target gene transcription	Yes;5mC
	Zfp281^336^		
	FOXA1^201^		
	EGR1^337^		
	NANOG^338^		
	STAT1^100^		
	TEAD^339^		
	FOXA2^340^		
	GFI1^341^	Recruit polycomb cofactors; Repress target transcription	No
Post-translational modification Enzymes	KAT8^342^	Recruit KAT8; Enhance gene transcription	No
	HDAC1^183^	Recruit HDAC1; Repress gene transcription	No
	SIRT1^343^	Enhance SIRT1 deacetylase activity	No
RNA-binding proteins	Lin28A^344^	Recruit TET1 to specific DNA sites; Enhance target gene transcription	Yes;5mC
	NONO^345^		
	FXR1^101^		
LncRNA	AC092723.1^346^	Recruit TET1 to specific DNA sites; Enhance target gene transcription	Yes;5mC
Others	RBAP46^347^	Recruit TET1 to specific DNA sites; Enhance target gene transcription	Yes;5mC
	SIN3A^348^		
	GADD45A^349^		
	GADD45B^350^		
	SUZ12^351^	Recruit TET1 to specific DNA sites; Enhance 5hmC formation	Yes;5mC
	QSER1^352^	Recruit TET1 to specific DNA sites; Repress DNMT3-mediated de novo methylation	No

**Table 2:** Different types of TET2 binding proteins and their functions

Type	Name[reference]	Effects	TET2 catalytic dependent; substrates
Transcription factors	PU.1,^121^ NANOG,^117^ PRDM14,^353^ E2A,^354^ SALL4A,^355^ RUNX1,^356^ CEBPA,^124^ KLF4,^124^ TFCP2L1,^124^ PML,^123^ BATF,^357^ ZSCAN4,^358^ EGR2,^359^ MAFB,^360^ FOXP1,^361^ Glucocorticoid receptor,^360^ HNF4α,^127^ RUNX2^362^	Recruit TET2 to specific DNA sites; Enhance target gene transcription	Yes;5mC
	IκBζ^187^	Allow TET2 to recruit HDAC2; Repress gene transcription	No
Post-translational modification Enzymes	OGT^363^	Allow TET2 to recruit H3K4 methyltransferases; Induce transcriptional activation	No
	OGT^364^	Stabilize TET2 association with chromatin	N/A
	OGT^365^	Promote GlcNAcylation of histone; Induce transcriptional activation	No
	HDAC2^187^	Recruit HDAC2; Repress gene transcription	No
	HDAC1/2,^222^ P300^222^	Regulate TET2 protein stability	N/A
	SIRT1^254^	Deacetylate TET2; Enhance its catalytic activity	N/A
	AMPK^223^	Phosphorylate TET2; Stabilize TET2 protein	N/A
	CRL4^227^	Ubiquitylate TET2; Regulate its stability	N/A
	JAK2^366^	Phosphorylate TET2; Enhance TET2 activity	N/A
	USP15^225^	Deubiquitinate TET2; Suppress TET2 activity	N/A
DNA methyltransferases	DNMT1^222^	Promote TET2 protein stability	N/A
RNA-binding proteins	PSPC1^192^	Allow TET2 to recruit HDAC1/2; Repress MERVL transcription	No
		Allow TET2 to oxidize m5C of MERVL; Facilitate the degradation of its transcripts	Yes; m5C
Others	IDAX^116^	Promote TET2 protein degradation through caspase activation	N/A
	VprBP^224^	Stabilize TET2 association to chromatin	N/A
	Vpr^227^	Promote TET2 ubiquitination degradation	N/A
	SNIP1^126^	Guide TET2 to particular DNA loci; Enhance target gene transcription	Yes;5mC
	CXXC5^367^	Upregulate transcription of target gene	Yes;5mC
	PROSER1^368^	Promote TET2 O-GlcNAcylation; Stabilize TET2 protein	N/A

*N/A* not applicable

**Table 3:** Different types of TET3 binding proteins and their functions

Type	Name[reference]	Effects	TET3 catalytic dependent; substrates
Transcription factors	FOXA2^138^	Recruit TET3 to specific DNA sites; Enhance target gene transcription	Yes;5mC
	TRα1^369^	Stabilize TRα1 protein	No
	HNF4α^370^	Recruit TET3 to specific DNA sites; Enhance 5hmC formation	Yes;5mC
Post-translational modification Enzymes	HDAC1^371^	Recruit HDAC1; Repress gene transcription	No
	OGT^372^	Catalyze TET3 O-GlcNAcylation; Promote cytoplasmic localization	N/A
Others	PGC7,^140^ SMCHD1^373^	Suppress TET3 activity	N/A
	glutamate dehydrogenase^235^	Enhance TET3 activity	N/A
	REST^63^	Recruit TET3 to specific DNA sites; Enhance target gene transcription	Yes;5mC
	SIN3A^371^	Recruit SIN3A; Repress gene transcription	No
	GSE^374^	Recruit TET3 to specific DNA sites; Enhance 5hmC formation	Yes;5mC

*N/A* not applicable

### Classical functions of TETs

#### TET1

Studies have shown the important role of TET1 in physiological functions, including development.^70^ The deficiency of TET1 lowered female germ-cell numbers by controlling meiosis through mediating related-gene DNA demethylation,^71^ while TET1 loss was dispensable for mice embryonic and postnatal development.^72^ However, acute deletion of TET1 caused a significant decrease of 5hmC levels and impaired embryonic stem cell identity,^73^ possibly because a long-term chronic reduction in TET1 led to homeostatic compensation.^73^ TET1 is also essential for intestinal stem cell functions in vivo^74^ and implicated in dynamic changes of DNA methylation during the maturation of fetal intestinal epithelial organoids in vitro.^75^ Epigenetic programming by catalytic-dependent TET1 is implicated in liver regeneration^76^ and remyelination in mouse brain.^77,78^ TET1 is involved in regulating iron homeostasis by demethylating the promoter of *RNF217* and this ubiquitin ligase is responsible for the degradation of iron exporter ferroportin.^79^ TET1-deficient mice exhibit impaired spatial learning and memory.^80^ Findings also support the roles of TET1 in promoting pluripotent stem cell induction.^81–84^ In addition, TET1 is required in the reprogramming of fibroblasts to dopaminergic neurons.^85^

Abnormal expression of TET1 is associated with many diseases.^86–92^ Loss of TET1 led to B cell malignancy in aged mice, partly due to aberrant DNA-hypermethylation,^93^ although *TET1* acted as an oncogene in *MLL*-rearranged leukemia.^94^ Additionally, insufficient TET1 was implicated in pulmonary arterial hypertension.^95^ Interestingly, overexpression of TET1 promoted cholangiocarcinoma progress via proliferative and anti-apoptotic signaling pathways,^96^ while insufficient TET1 accelerated intestinal tumorigenesis.^97^ Besides, high expression of TET1 appeared to be involved in polycystic ovary syndrome with hypomethylation signatures.^98^

Protein interactions enable rapid regulation and represent an important regulation in TETs functions, which allow precise modifications in specific DNA loci timely.^99^ For example, TET1 interacted with STAT1, contributed to the demethylation of *IRF1* promoter and transcriptional upregulation of PD-L1, to drive tumor immune evasion.^100^ In addition, interestingly, FXR1, an m6A reader, guided TET1 to specific genomic loci near m6A RNA to result in DNA demethylation, revealing a novel regulation between RNA modification and DNA demethylation.^101^ Similar models have been supported by many findings, which are summarized in Table 1.

#### TET2

Unlike *TET1* and *TET3*, *TET2* mutations with high frequency are identified in hematologic malignancies.^102–105^ Thus, the relationship between *TET2* mutations and overall survival has been investigated. Evidence showed that the patients with *TET2* mutations had worse overall survival compared with the patients with wild-type TET2 in 93 patients with AML.^38^ However, other studies showed no survival association in 111 patients with de novo AML^106^ and in a cohort of 247 patients with secondary AML.^107^ Therefore, the significance of *TET2* mutations in AML prognosis remains to be elucidated. The effects of *TET2* mutations on its functions, such as enzymatic activity and the ability of binding other proteins, and potential confounding variables should be considered. Further studies suggest that TET2 works as a tumor suppressor.^108–112^ Interestingly, findings also reveal tumor-promoting roles of TET2.^113,114^ For example, TET2 maintained the immunosuppressive-related gene expression in tumor-associated macrophages.^115^

As TET2 does not contain the CXXC domain, this raises a question of how TET2 is bound with the chromatin? One reasonable hypothesis is that, IDAX, originating from the ancestral TET2 CXXC domain, mediates its chromatin recruitment. Indeed, biochemical studies demonstrated that IDAX could bind to TET2 directly, suggesting that IDAX was able to recruit TET2 to DNA.^116^

In addition to IDAX, some other TET2-binding proteins have been identified by biochemical studies (Table 2). For instance, TET2 interacted with NANOG and played an important role in the establishment of pluripotency in a NANOG-dependent manner.^117^ EBF1, a transcription factor, had also been identified as a TET2-binding protein by co-immunoprecipitation of TET2 and EBF1. Importantly, sequence analyzing revealed that these two proteins were enriched in a proportional way, implying that TET2, without a CXXC DNA-binding motif, exploited a DNA-binding protein, such as a transcription factor, to regulate sequence-specific DNA demethylation.^118^ This idea was reinforced by the interaction of TET2 with WT1.^119,120^ Further studies revealed that mutations of *TET2* and *WT1* were mutual exclusively in AML, and WT1 guided TET2 to a specific DNA sequence, leading to the demethylation and activation of WT1-target genes.^120^ Many following studies supported this model, in which a DNA-binding factor recruited TET2 to a specific DNA sequence and regulated the expression of this gene in certain contexts.^121–127^ For instance, we found that the transcription factor HNF4α could recruit TET2 to *FBP1* promoters, resulting in the increase of FBP1 expression, to suppress the tumor growth.^127^ These models relied on the oxygenase activity of TET2.

#### TET3

As a member of the TET family, the main role of TET3 is implicated in demethylation in many biological processes such as zygote formation,^128–131^ embryogenesis,^132^ axon regeneration,^133^ and synaptic transmission.^134^ For example, TET3-mediated DNA demethylation is necessary for liver tissue maturation *via* proper hepatocyte gene expression.^135^ In addition, TET3 deficiency induced by mutations is associated with abnormal growth and intellectual disability,^136^ indicating the fundamental role of TET3 in development. In adult mice, TET3 ablation is associated with anxiety-like behaviors, although the molecular mechanisms remain to be explored.^137^

Interestingly, hepatic TET3 was recruited to the promoters of the fetal version of HNF4α by FOXA2, contributing to high expression of HNF4α transcription by promoter demethylation, and this process impaired glucose homeostasis due to HNF4α-mediated gluconeogenesis activation. Thus, these findings linked TET3 to type-2 diabetes.^138^ In addition, insufficient demethylation of several insulin secretion genes, owing to the maternal inheritance of oocyte TET3 insufficiency, contributed to glucose intolerance.^139^ These findings demonstrated the distinct roles of TET3 in certain contexts. Similar to TET2, binding partners are involved in TET3 function regulation (Table 3). For example, PGC7 interacted with TET3 and suppressed TET3 enzymatic activity to protect DNA methylation at imprinting loci during early embryogenesis,^140^ although PGC7 bound to H3K9me2 to block the TET3-mediated conversion of 5mC to 5hmC.^141^

#### TET1/2/3

Furthermore, in some biological contexts, TETs cooperate with each other to orchestrate specific functions. For instance, TET1 and TET2 are involved in pluripotent reprogramming and imprint erasure induced by cell fusion,^142^ erasure of 5mC in mouse primordial germ cells,^143^ pre-mRNA alternative splicing,^144^ maintaining stem cell identity,^145^ reprogramming to recover youthful DNA methylation patterns in aged mice^146^ and epigenetic reprogramming in offspring caused by maternal exercise.^147^ Binding proteins are required for desired functions in some cases. For example, upon TGF-β and IL-2 signaling, TET1 and TET2, recruited by SMAD3 and STAT5, bound to and subsequently demethylated *FOXP3* promoter to maintain immune homeostasis.^148^ Similarly, to main bone homeostasis, both TET1 and TET2 were required for demethylating promoters of *P2RX7*.^149^ Additionally, TET1 and TET3 are associated with cerebellar circuit formation^150^ and CD4 expression in peripheral T cells.^151^

TET2 and TET3 are required for Treg cell stability and immune homeostasis,^152^ and improve Treg cell efficacy by increasing the stability of FOXP3.^153^ TET2 and TET3 acted as recruiters of HDACs to suppress CD86 and prevent autoimmunity.^154^ Findings also reveal the roles of TET2 and TET3 in embryonic heart development^155^ and in regulating proper development and maturation of invariant natural killer T cells.^156^ Knockdown of TET2 led to hyper-proliferation of erythroid progenitors, whereas knockdown of TET3 impaired terminal erythroid differentiation. These findings revealed distinct roles of TET2 and TET3 in the regulation of human erythropoiesis.^157^ Furthermore, the deletion of TET2 and TET3 led to aggressive myeloid cancer in mice.^158^ Mice with TET2 and TET3 double knockout in mature B cells developed B cell lymphoma, which can be delayed upon DNMT1 deletion,^159^ suggesting the importance of proper methylome.

TET1, TET2, and TET3 are required for somatic cell reprogramming of fibroblasts to pluripotency,^160^ telomere homeostasis,^161^ and early body plan formation.^162^ Human embryonic stem cells (hESCs) with triple-knockout of TET1, TET2, and TET3 exhibited prominent bivalent promoter hypermethylation, suggesting the role of TETs in maintaining hypomethylation at bivalent promoters to ensure proper lineage-specific transcription during differentiation.^163^ In mESCs, TETs tended to increase demethylation rates at enhancer elements.^164^ Distinct roles of TETs in regulating 5hmC formation, DNA demethylation, and gene expression are also explored in cancer cells.^165^

The overlapping roles of TETs have been explored due to their similar enzymatic activity. Mice with loss of either TET1^72^ or TET2^166^ are viable, while most TET1/2 double knockout mice die perinatally,^167^ suggesting that deletion of the individual TET gene can be compensated by other TETs. Interestingly, TET3 knockout leads to neonatal lethality,^128^ indicating the unique role of TET3 that could not be compensated by the other TETs. Thus, the overlapping roles of TETs in certain contexts have not yet been fully established. In addition, to understand the TETs functions in vivo, mouse models with gene constitutive or conditional knockout have been generated, some of which are summarized in Table 4.

**Table 4:** Representative mouse models with TETs loss of function

TETs loss of function	Major phenotypes	References
*TET1* knockout	B cell malignancies	^93^
*TET2* knockout	CMML-like	^375^
*TET3* knockout	Neonatal lethality	^128^
*TET1/2* double knockout	B-lymphoid malignancies	^376^
*TET1/3* double knockout	Embryonic lethality	^377^
*TET2/3* double knockout	Myeloid leukemia	^158^
*TET1/2/3* triple knockout	Embryonic lethality	^162^

*CMML* chronic myelomonocytic leukemia

#### 5hmC

TETs-mediated 5hmC formation appears to be an epigenetic mark, although the physiological significance has not been fully elucidated.^168–170^ The 5hmC acquisition occurred in mouse, rabbit, and bovine zygotes,^171^ indicating that the mark was conserved in these mammalian species. MBD3, required for pluripotency in ESCs,^172^ preferred to binding 5hmC-containing probes rather than 5mC-containing probes and regulated the expression of genes with 5hmC modifications in ESCs.^173^ In addition, the acquisition of 5hmC by TET1 in enhancers was associated with enhancer activation,^174^ implying that 5hmC represented a signal mark rather than an intermediate. The idea was supported by the role of 5hmC in germline reprogramming^175^ and in drug addiction.^176^ Interestingly, particular 5hmC acquisition by cocaine lasted at least one month in mouse nucleus accumbens.^176^ Besides, TET1-mediated 5hmC deposition was also implicated in osteoarthritis.^177^

Interestingly, 5hmC formation is not required for the loss of paternal 5mC in early mouse zygotes,^178^ further supporting the fascinating and mysterious role of 5hmC, not just the demethylation intermediate. 5hmC modifications have been reported to affect protein binding,^179^ and consistently, 5hmC might recruit a chromatin-modifying complex to suppress transcription.^180^ 5hmC formation caused by TET3, prevented spurious transcription, which was critical for maintaining transcriptional fidelity in the lung.^181^

### Moonlighting functions of TETs

Overexpression of either TET1 or catalytic-death TET1 impaired long-term memory in mice, suggesting the catalytic-independent function of TET1.^182^ Furthermore, TET1 acted as an epigenetic suppressor of thermogenesis in beige adipocytes largely independent of its catalytic activity. Specifically, TET1 interacted with HDAC1 to suppress key thermogenic gene transcription by reducing histone acetylation.^183^ Consistently, catalytic-independent functions of TET1 in silencing developmental genes by regulating H3K27 modifications,^184^ supported that TET1 acted as an interaction hub for recruiting different chromatin-modifying complexes in a non-catalytic manner.^185^ Besides, the non-catalytic function of TET3 in transcriptional repression of *SNRPN* by binding to *SNRPN* promoter, was critical for the maintenance of adult neural stem cell state.^186^

Apart from the ability of DNA oxygenase, studies also unveiled that TET2 could reduce inflammation by repressing IL-6, which is independent of its role in converting DNA 5mC to 5hmC. Specifically, TET2, binding with IκBζ, recruited HDAC2 to promote histone deacetylation, which led to the repression of IL-6 at the transcription level. These findings provided a TET2 enzymatic-independent function in repressing specific gene transcription.^187^

To explore the enzymatic versus nonenzymatic roles of TET2 in hematopoiesis, Ito et al. performed a comparative analysis of *TET2* catalytic mutant mice and *TET2* knockout mice. This study found that mice with non-catalytic TET2 mainly developed myeloid malignancies, while mice with complete loss of TET2 developed both myeloid and lymphoid disorders, supporting the unique non-catalytic role of TET2 in the hematopoietic stem and progenitor cell homeostasis.^188^

Interestingly, besides its well-known function in regulating the modification of DNA, TET2 possessed the activity of oxidating 5mC RNA (m5C) into 5-hydroxymethylcytidine (hm5C). Fu et al. found that the catalytic domain of TET2 could induce the formation of hm5C in HEK293T cells. Considering hm5C accounting for approximately 0.02% of total m5C RNA in tumor samples, this implied the involvement of TET2 in RNA biology.^189^ Consistently, a study in Drosophila showed that TET protein was involved in the formation of hm5C.^190^ This study also mapped the distribution of hm5C and revealed hm5C located in coding sequences of many gene transcripts. Importantly, hm5C favors mRNA translation.^190^ However, the biofunction of hm5C in mammalian RNA is largely unknown until Shen et al. discovered that TET2 was involved in RNA stability.^191^ These findings uncovered that TET2, depending on its enzymatic activity of mRNA oxidation, promoted pathogen infection-associated myelopoiesis. Specifically, TET2 mediated oxidation of SOCS3 m5C, which led to ADAR1 binding and destabilizing SOCS3 mRNA and consequently repressed SOCS3 expression.^191^ Meanwhile, by the TET2 interactome in mouse ESCs, Guallar et al. identified that paraspeckle component 1(PSPC1), an RNA-binding protein, could bind to TET2 and this complex recruited HDAC1/2 for repression of *MERVL* transcription independent of TET2 catalytic activity. More importantly, this study further found that TET2, recruited by PSPC1, catalyzed hm5C modification of *MERVL* RNAs, facilitating the degradation of *MERVL* transcripts, and thus provided a new paradigm for TET2-mediated post-transcriptional silencing of the specific gene. Notably, PSPC1 and its RNA-binding domains are essential for TET2 function in regulating *MERVL* by both transcriptional and post-transcriptional mechanisms.^192^ Interestingly, using a proteomics approach, Huang et al. discovered PSPC1 also bound to TET1 for bivalent gene regulation in formative pluripotency independent of the catalytic activity of TET1.^193^ Additionally, TET2 has been shown to function in ESC differentiation by reducing the pluripotency-related mRNA stability, caused by TET2-mediated hm5C.^194^ Notably, this study confirmed that TET2 contained an RNA-binding domain, which had been identified by a proteomic approach in a previous study.^195^

In addition to its oxidation of mRNA, recently, He et al. found that TET2 could convert m5C into hm5C in tRNA, subsequently affecting tRNA fragment levels.^196^ Meanwhile, m5C oxidation in tRNA mediated by TET2 facilitated translation.^197^ These findings linked TET2-mediated tRNA modification to tRNA processing and mRNA translation,^196,197^ unveiling novel roles of TET2 in gene regulation at multiple levels. Additionally, findings revealed that TET1/2 could oxidize T to 5hmU in mESCs.^198^

**Fig. 4:**
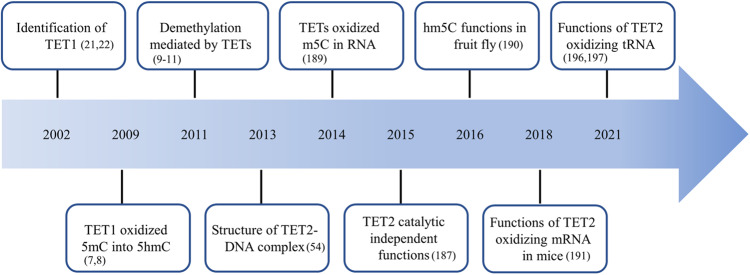
The timeline of key discoveries in basic research of TETs

In this part, the interaction with various binding proteins stably and transiently mainly affects TETs location, including recruiting TETs to specific sites, allowing TETs to recruit other proteins, and stabilizing TETs association with DNA. Besides, TETs are capable of oxidizing both DNA and RNA. Understanding the characteristics of TETs might provide key insights into epigenetic editing, such as DNA demethylation and mRNA modification. Here, we summarize major discoveries in the history of TETs over time (Fig. 4).

## TET function regulators

**Fig. 5:**
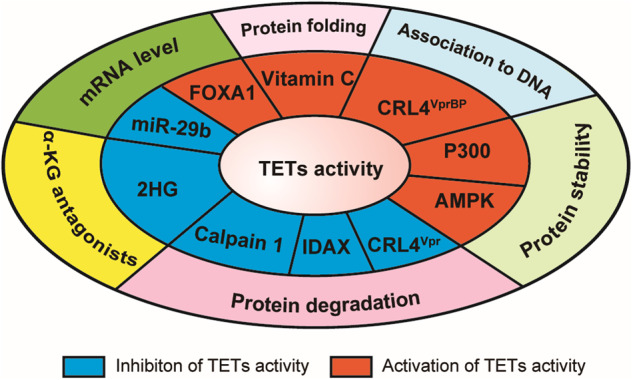
Primary factors in positive(red) and negative(blue) regulation of TETs activity and the outmost layer described the involved mechanism. FOXA1,^201^ Vitamin C,^238^ CRL4(VprBP),^224^ P300,^222^ and AMPK^223^ enhanced TETs activity, while Vpr,^227^ IDAX,^116^ Calpain 1,^228^ 2HG^232^ and miR-29b^207^ decreased TETs activity

Numerous studies have identified the factors in regulating TETs function, including transcription factors, microRNAs, post-translational modifications, and small molecules in different levels, as summarized in Fig. 5.

### Transcriptional level

Pluripotency-associated transcriptional factors, such as MYC and NANOG, regulated *TET1* expression in hESCs.^199^ *TET1/2* was regulated by Oct4 and SOX2.^200^ Interestingly, FOXA1 not only transcriptionally regulated *TET1*, but also interacted with TET1 to mediate DNA demethylation of its targeted enhancers.^201^ Besides, STAT3/5 transcriptionally activated *TET1* expression in AML^202^ and P53 positively regulates *TET1/2* transcription in mESCs.^203^ SIN3A increased *TET1* and *TET2* mRNA expression in human pulmonary arterial smooth muscle cells.^95^ Transcriptional suppression was also identified in the regulation of TETs. NF-κB-mediated repression of *TET1* transcription was uncovered in basal-like breast cancer.^204^ TET3, transcriptionally repressed by the nuclear receptor TLX, acted as a tumor suppressor in glioblastoma.^205^

### microRNAs

Using the TCGA database, the miR-29 family were predicted to regulate DNA demethylation by potentially targeting TET1.^206^ Indeed, miR-29b directly targeted and repressed *TET1* to promote the mesendoderm lineage formation^207^ and the miR-19b/TET1 axis could be utilized in attenuating osteoarthritis progression.^208^ Downregulation of TET1 also has been reported by miR-494 in hepatocellular carcinoma tumors^209^ and by miR-191 in intrahepatic cholangiocarcinoma,^210^ respectively.

Multiple studies have also demonstrated that microRNAs are involved in downregulating TET2 expression. Biochemistry studies discovered that miRNA-22 could directly bind to TET2 mRNA and negatively regulate TET2 expression, which contributed to myelodysplastic syndrome and hematological malignancies.^211^ miRNA-29a could also downregulate TET2 expression.^212^ A thorough analysis of TET2-targeting miRNA by a high-throughout 3’UTR screen, identified extensive miRNAs such as miRNA-29b and miRNA-101, inhibiting TET2 expression and these miRNAs regulated malignant hematopoiesis.^213^ Further studies identified that TET2 was under control of miRNA-Let7,^214^ miRNA-210^215^ miRNA-144-3p,^216^ miRNA-142-3p,^217^ miRNA-26a^218^ and miRNA-10b-5p.^219^ Besides, TET3 as a miR-150 target was associated with the generation of non-classical monocytes.^220^

### Post-translational modifications

Post-translational modification is also a key process in regulating TETs functions. Bauer et al. found that phosphorylation and O-GlcNAcylation existed in TET2 protein modification,^221^ indicating that complex modification modulated TET2 functions in different conditions. Indeed, P300-mediated acetylation of conserved lysine residues enhanced TET2 stability, and increased its ability to target chromatin, which reduced aberrant DNA methylation, and thereby protected against abnormal DNA methylation induced by DNA damage.^222^ Additionally, AMP-activated kinase catalyzed the phosphorylation of TET2 at serine 99, which increased the stability of TET2. While the phosphorylation of TET2 was inhibited under hyperglycaemic conditions such as diabetes, consequently decreasing TET2 levels.^223^ Monoubiquitylation of TET2 at lysine 1299 mediated by VprBP facilitated TET2 association to chromatin, whereas mutation of TET2 at 1299 blocked its interaction with VprBP and decreased its association with DNA.^224^ Interestingly, the K1299-linked monoubiquitylation of TET2 could be removed by USP15, decreasing TET2 association to DNA.^225^ Additionally, phosphorylation of TET3 by CDK5 caused lower binding affinity to histone variant H2A.Z. and contributed to higher level of 5hmC at *BRN2* promoter to activate BRN2 expression during neuronal differentiation.^226^

### Protein degradation

Surprisingly, besides the CRL4 E3 ligase mediated TET2 monoubiquitylation promoted TET2 association to chromatin, HIV-1 derived Vpr hijacked CRL4, and this E3 ligase preferred to catalyze polyubiquitylation of TET2, accordingly promoting TET2 degradation to sustain IL-6 expression and facilitate viral replication.^227^ Unexpectedly, IDAX, the TET2-binding protein, promoted TET2 degradation in a caspase activation-dependent manner.^116^ With different proteolytic pathway inhibitors, calpains were identified to be involved in TET2 protein regulation. Specifically, calpain 1 was implicated in the degradation of TET2 in ESCs, leading to skewing lineage expression.^228^

### Small molecules

As α-KG is required to maintain the oxygenase activity of TETs, it is plausible that 2HG, generated by the reduction of α-KG catalyzed by IDH enzyme mutants,^229^ might disrupt TETs function.^230^ Indeed, biochemistry studies demonstrated that mutant IDH decreased TET2-mediated 5hmC levels.^231^ Consistently, structure analysis revealed that 2HG occupied the site of α-KG in protein conformational space, suggesting that 2HG served as a competitive inhibitor of α-KG-dependent enzyme activity, including TET2.^232^ In addition to 2HG, succinate and fumarate were also identified to act as α-KG antagonists, which inhibited TET2 dioxygenase activity.^233^ Recently, Chen et al. found that itaconate was also a TET2 dioxygenase inhibitor through the competition with α-KG to interact with TET2, resulting in dampening inflammatory responses.^234^ Besides, the nuclear glutamate dehydrogenase interacted with TET3 to supply TET3 with αKG and increased its demethylation activity in neurons.^235^

Previous studies have revealed that vitamin C could upregulate the activity of some α-KG-dependent dioxygenases, suggesting that vitamin C might be involved in the modulation of TETs activity. Indeed, vitamin C could enhance TET2 activity and subsequently increase 5hmC levels in ESCs.^236,237^ Yin et al. found that vitamin C, but not other reducing chemicals such as NADPH and vitamin E, was a unique activator of TET dioxygenases.^238^ It is possible because vitamin C was capable of binding to the catalytic domain of TET proteins, facilitating protein folding, and accelerating oxidation reactions.^238^ The idea, that vitamin C acting as a TET agonist, was reinforced by a series of further studies.^239–245^ Notably, TET2 deficiency presented in aberrant self-renewal and leukemia progression, which can be blocked by treatment with vitamin C, suggesting that vitamin C treatment might be beneficial to patients with leukemia.^246^ Specifically, vitamin C restored TETs function and drove the expression of related genes.^246^

Aside from metabolites, Thienpont et al. found that the activity of TET2 was reduced under hypoxic conditions, leading to DNA-hypermethylation.^247^ Oxygen levels determined the activity of TET1 in ESCs.^248^ Redox-active quinones promoted the production of 5hmC by TETs.^249^

Artificial inhibitors and activators of TETs have also been explored. By screening strategy, a small molecule compound, C35, was identified as a TETs inhibitor. Notably, this compound specifically blocked TETs catalytic activities without abolishing TETs complexes.^250^ Bobcat339, one of synthesized cytosine derivatives, inhibits TET1 and TET2 activity.^251^ A small molecule, TETi76, inhibits TETs specifically.^252^ Interestingly, Nickel (II) exhibits inhibition to TETs enzymatic activities by replacing the cofactor Fe (II) of TETs.^253^ Additionally, SRT1720, a SIRT1 agonist, by deacetylating TET2, significantly increases TET2 activity.^254^

Together, similar to other genes, TETs can be regulated at multiple levels, including post-transcriptional and post-translational regulation. Furthermore, it can be modulated by small molecules involved in its enzymatic reaction. This ensured the fine-tuning of TETs enzymatic activity in response to external cues.

## Targeted therapy and clinical trials

Given the various roles of TETs in biological processes, it comes as no surprise that it has been proposed as an important therapeutic target for diseases such as cancer.^113,114,252,255,256^ For example, vitamin C, by improving TETs activity, allows leukemia cells to be more sensitive to PARP inhibitors.^246^ Interestingly, cells with *TET2* mutations, possibly heavily relying on compensatory roles of TET1/3, showed more vulnerable to TETs inhibitors compared with normal ones. These findings provide a new therapeutic strategy for selective targeting of cells bearing TET2 mutations.^252^ 5-azacytidine, a DNA demethylating agent, shows higher cytotoxicity in *TET2*-silenced cells, probably due to the hypermethylation pattern caused by the loss of TET2.^256^ In addition, C35, a selective TETs inhibitor, promotes somatic cell reprogramming.^250^ As a robust TET2 activator, clinical trials are investigating the effects of Vitamin C on hematologic malignancy patients with *TET2* mutations (NCT03397173; NCT03433781). Of note, the antitumor effects of vitamin C has been studied for a long time; however, its efficacy against cancers have not been established by clinical trials, possibly because of the complex mechanisms of action of vitamin C.^257–262^ As a new target, the role of TET2 enzymatic activity enhanced by vitamin C in patients with hematological malignancies remains unclear. Besides, clinical trials evaluating the contribution of vitamin C-mediated upregulating TET2 enzymatic activity in solid tumors are urgently required. Notably, high concentrations of vitamin C administration with or without anticancer drugs have not shown serious adverse effects in clinical trials, suggesting that vitamin C is a drug with low toxicity.^263–267^ Therefore, vitamin C might be a promising anticancer treatment option for cancer patients with dysfunctions of TET2 in the future.

## Detection of 5hmC

**Fig. 6:**
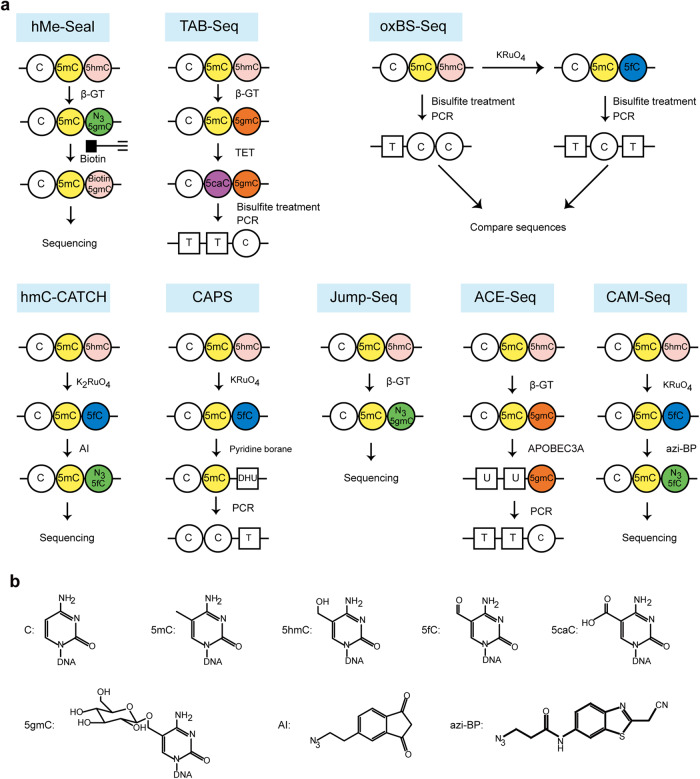
Representative schematic diagrams of 5hmC detection approaches. **a** Schematic diagrams of hMe-Seal,^296^ TAB-seq,^297^ oxBS-seq,^298^ hmC-CATCH,^299^ CAPS,^301^ Jump-Seq,^302^ ACE-Seq,^303^ and CAM-Seq.^304^ **b** Structural formula of representative molecules

5hmC plays distinct epigenetic roles in mESCs.^268,269^ In addition, aberrant levels of 5hmC are associated with various cancers.^270–278^ Furthermore, 5hmC signatures in circulating cell-free DNA can be used as biomarkers for cancer diagnosis.^279–283^ Together, mapping the distribution of 5hmC in a genome is important not only to elucidate its biology, such as functions in development, but also to use it for clinical potential.^284–288^ In this section, representative approaches for detecting 5hmC with or without bisulfite treatment are discussed (Fig. 6).

### hMeDIP

To investigate the global distribution of 5hmC, anti-5hmC antibodies were utilized to capture 5hmC DNA from genomic DNA followed by sequencing, and this approach was named as hMeDIP.^289–291^ This method is cost-effective and widely used. However, the biggest limitation of this method is the quality of anti-5hmC antibodies. To solve the problem caused by using antibodies of different production batches, Robertson et al. developed a novel 5hmC detection method, based on the selective glycosylation of 5hmC treated with β-glucosyltransferase. This β-glucosyl-5-hydroxymethylcytosine-containing DNA could be efficiently and specifically captured by J-binding protein 1. After enriching 5hmC, further analysis could be performed, such as qPCR and sequencing.^292,293^ Likewise, 5hmC was converted to cytosine-5-methylenesulfonate (CMS) upon sodium bisulfite treatment, and then the CMS-specific antiserum was used to capture CMS-containing DNA fragments for further analysis.^294,295^

### hMe-Seal

Bisulfite treatment could lead to significant degradation of DNA, and therefore bisulfite-free methods were developed for limited DNA samples. For example, β-glucosyltransferase could convert 5hmC to β-glucosyl-5-hydroxymethylcytosine (5gmC) in the presence of UDP-Glu. The 5hmC can be labeled with an azide group using the modified UDP-Glu with the azide. This allowed biotin moiety containing an alkynyl group to link to 5hmC using click chemistry, followed by affinity enrichment and sequencing.^296^

### TAB-Seq

In 2012, Yu et al. developed a TET-assisted bisulfite sequencing approach, named as TAB-Seq, which enabled the detection of genomic 5hmC sites at single-base resolution. Specifically, 5mC could be oxidized to 5caC with TET proteins and the 5caC could subsequently be deaminated to form U by bisulfite treatment, while the glucosylated-5hmC was protected from TET oxidation and bisulfite deamination and therefore was identified as C. This method allowed discriminating 5hmC from 5mC, in contrast with traditional bisulfite sequencing.^297^

### oxBS-Seq

Meanwhile, Booth et al. also developed a method of quantitatively mapping 5hmC distribution at single-base resolution, known as oxidative bisulfite sequencing (oxBS-Seq). This approach utilized potassium perruthenate to selectively oxidate 5hmC to 5fC that was subsequently converted to U by bisulfite treatment, while 5mC was not oxidized by potassium perruthenate and still detected as C. This method enabled the determination of the amount of specific 5hmC sites by subtracting the readout of traditional bisulfite sequencing.^298^

### hmC-CATCH

Similar to oxBS-Seq, potassium ruthenate was used to convert 5hmC to 5fC, which was further selectively modified with an azido, and this adduct was identified as T during PCR. Therefore, the C-to-T transition was regarded as the readout of 5hmC. Additionally, the azido group rendered it easily for enrichment and sequencing.^299^

### CAPS

Similar to TAB-seq, TETs were employed to convert both 5mC and 5hmC to 5caC, and pyridine borane was subsequently used to convert 5caC to dihydrouracil, that was read as T during PCR. This modified C-to-T transition allowed whole-genome detection of 5mC and 5hmC at single base-level resolution. In contrast, glucosylated-5hmC was inert to TET oxidation and borane reduction, and thus 5mC sites could be analyzed specifically.^300^ Accordingly, the amount of 5hmC sites could also be determined by comparing the readouts with or without β-glucosyltransferase treatment at the first step. Alternatively, TET proteins could be replaced by potassium perruthenate to selectively oxidate 5hmC to 5fC, allowing specifical sequencing of 5hmC.^301^

### Jump-seq

A new strategy, called Jump-seq, was developed by Hu et al. for detecting 5hmC without sequencing the whole genome at nearly a single-base resolution. This method took advantage of selectively labeling 5hmC with a glucose moiety carrying an azide group, followed by linking a hairpin DNA with an alkyne group. 5hmC positions could be deduced by the connection between genomic DNA sequence and the hairpin sequence after primer extension.^302^

### ACE-seq

APOBEC3A-based 5hmC sequencing method, named ACE-seq, has been developed without bisulfite treatment at single-base resolution. 5hmC was modified with glucose by β-glucosyltransferase and the glucose-modified 5hmC was inert to APOBEC3A, a DNA deaminase, whereas C and 5mC could be converted to U, yielding 5hmC identified particularly.^303^

### CAM-Seq

With a similar strategy, 5hmC was initially converted to 5fC by KRuO4. Then using azi-BP, a compound reported by the same group, 5fC was selectively labeled, rendering it matching with A and identified as T by PCR. Using this method 5hmC loci in genomic DNA could be analyzed at single-base resolution.^304^

As the findings of the important role of TET families in DNA modification, selective chemical labeling of the hydroxyl group of 5hmC is fast-growing to map the genome-wide distribution of 5hmC. Here, we summarize some characteristics of each method in Table 5. Of note, recently, nanopore sequencing technologies have shown a diverse range of applications, including 5hmC detection.^305,306^ In addition, hm5C could be detected by mass spectrometry.^307,308^ Like hMeDIP, hm5C-containing RNA could be captured by the anti-hm5C antibody followed by sequencing and this method was named as hMeRIP-seq.^190,194^

**Table 5:** Representative approaches for 5hmC detection

Name	Principle	Bisulfite-based	Minimal sample amounts(range)	Base resolution	Refs
hMeDIP	Enrich 5hmC DNA fragments by a 5hmC antibody	No	Microgram	No	^289–291^
hMe-Seal		No	Nanogram	No	^296^
TAB-Seq		Yes	Microgram	Yes	^297^
OxBS-Seq		Yes	Microgram	Yes	^298^
hmC-CATCH		No	Nanogram	Yes	^299^
CAPS		No	Nanogram	Yes	^301^
Jump-Seq		No	Nanogram	No	^302^
ACE-Seq		No	Nanogram	Yes	^303^
CAM-Seq		No	Nanogram	Yes	^304^

*β-GT* β-glucosyltransferase, *5gmC* β-glucosyl-5-hydroxymethylcytosine, *AI* an azido derivative of 1,3-indandion, *DHU* dihydrouracil, *TAB-Seq* Tet-assisted bisulfite sequencing, *oxBS-Seq* oxidative bisulfite sequencing, *hMe-Seal* 5hmC selective chemical labeling, *hmC-CATCH* chemical-assisted C-to-T conversion of 5hmC sequencing, *CAPS* chemical-assisted pyridine borane sequencing, *Jump-seq* 5hmC sequencing without sequencing the entire genome, *ACE-seq* APOBEC-coupled epigenetic sequencing, *CAM-Seq* chemical-assisted mismatch sequencing

## Demethylation editing tools

**Fig. 7:**
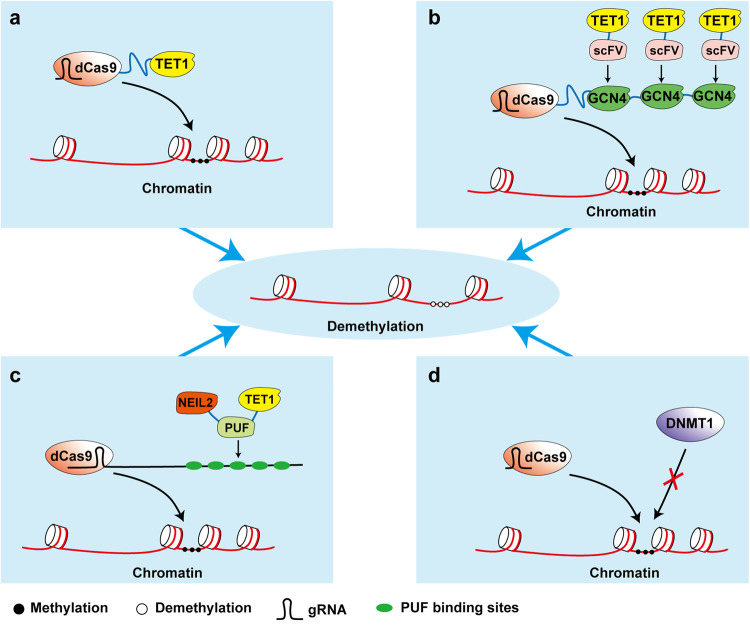
Representative working models of targeted demethylation. **a** With the gDNA, a DNA modification domain, such as TET1, fused to dCas9, led to the erasure of specific DNA methylation.^333^ **b** GCN4 repeats fused to dCas9, recruited many copies of an anti-GCN4 antibody (scFV)-fused TET1, to amplify demethylation efficiently.^322^ **c** Multiple effectors were used to increase the efficacy of demethylation. The modified gRNA with PUF binding sites, recruited protein fusions of PUF, TET1, and NEIL2 to particular DNA methylation sites. Among these, PUF were used for binding to the modified gRNA, and TET1 oxidated 5mC and NEIL2 worked as a DNA glycosylase to promote DNA demethylation.^324^ **d** Without tethering an effector such as TET1, only gRNA-dCas9 led to specific DNA demethylation, by sterically blocking DNA methyltransferase^331^

Dynamic regulation of DNA methylation and demethylation plays a critical role in many biological processes, including epigenetic memory, genomic imprinting, and development.^309–311^ Dysregulation of this process leads to many diseases such as autoimmune disorders and cancers.^312–314^ In addition, hypermethylation patterns are usually associated with gene silencing. Therefore, developing epigenetic editing tools allow us not only to modify the target locus to evaluate the consequences of epigenetic marks, but also to silence or activate the gene in specific contexts. The general idea of epigenetic editing is that an epigenetic writer or eraser is fused to a sequence-specific DNA-binding domain to rewrite the epigenetic marks in targeted loci or histone^315,316^(Fig. 7). In this part, we summarize TETs-based epigenetic editing tools (Table 6).

**Table 6:** Representative demethylation tools

Demethylation effectors	DNA-binding platform	References
TET1	TALE	^317,318^
TET1	dCas9	^319–323,325,326,333,334^
TET1	Reverse tetracycline transactivator	^327^
TET1, GADD45A, NEIL2	dCas9	^324^
TET2	Zinc fingers	^328,378^
TET3	dCas9	^329^
ROS1	dCas9	^330^
**-**	dCas9	^331^

*TALE* transcription activator-like effector

TET1-TALE-fused-based tools were developed for epigenetic editing,^317,318^ and successfully increased β cell replication, demonstrating a promising approach in therapeutic applications.^318^ Customized TALE repeat arrays worked as a platform for guiding TET1 to the DNA sequence of interest, therefore leading to the demethylation of targeted loci, and subsequently increasing the related gene expression.^317^

Additionally, engineered endonuclease-dead Cas9 (dCas9) could also be used as a linker, and recruited indirectly or fused directly to the designed effector domains, such as TET1, to modify the specific target in conjunction with gRNA.^319–323^ Furthermore, co-delivery of demethylation pathway-related proteins such as GADD45A and NEIL2, with dCas9-TET1, enhanced demethylation editing efficacy.^324^ Besides, the CRISPR/dCas9-based gene transcription activation system coupled with TET1, activated silenced genes through demethylating.^325,326^

In addition to dCas9, other DNA-binding domains worked as a target loci modification guider. For example, a synthetic fusion protein, carrying enzymatic domains of TET1 and reverse tetracycline transactivator, exhibited demethylation of Tet promoter, upon doxycycline treatment.^327^ Similarly, TET2 was fused to a DNA-binding domain to promote the demethylation of targeted loci, and thereby a TET2-based editing approach was developed.^328^ The engineered protein contained two core domains: TET2 for inducing DNA demethylation and zinc fingers for binding the ICAM-1 promoters.^328^

Other effectors could also be employed such as TET3 and ROS1. TET3 catalytic domains, fused to dCas9, could produce 5hmC formation.^329^ Plants DNA demethylases such as ROS1 could replace TET1 to induce demethylation.^330^ Interestingly, simple CRISPR/dCas9 and gDNA without tethering any other enzymes appeared to demethylate target loci efficiently largely due to steric blockage of DNA methyltransferase.^331^

Methylation editing tools have shown great potential in clinical research and treatment. Model mice with Silver-Russell syndrome has been successfully generated by TET1-dCas9 based system.^332^ TET1-based DNA methylation editing could restore the expression of FMR1 by demethylating its promoter, supporting the potential application of epigenome editing in fragile X syndrome treatment.^333^ Similarly, TET1-dCas9 mediated demethylation of the MECP2 promoter, rescued Rett syndrome neurons.^334^ Thus, precise and efficient epigenetic editing tools would provide new insights into the functions of the specific DNA modification locus temporal-spatially.

## Summary

Here we review the remarkable findings in understanding the function of TETs in modifications of DNA and RNA, and summarize recent advances in the detection of 5hmC and DNA demethylation editing tools. Despite the formation of oxidation products (5hmC, 5fC, and 5caC) and the mechanism of active DNA demethylation have been characterized, some questions have yet to be answered. First, the significance of 5hmC needs to be delineated. Second, in addition to oxidating DNA, recent studies have also demonstrated that TET2 is capable of oxidating RNA. It is still not well-defined what factors determine TET2 in choosing oxidating DNA or RNA. Third, regardless of containing DNA-binding domain, all TETs appear to be recruited to specific DNA sequences by their binding partners. It is worth to further explore how to modulate the binding of TETs to its target DNA sequences in various biological processes. Fourth, loss of function mutations of *TET2* are frequently identified in blood malignancies, whereas mutations of *TET2* are uncommon in solid tumors. However, significant downregulation of TET2 activity is observed in many solid tumors. The underlying mechanisms are still not clear and require to be explored for the diagnosis and therapy of cancers. We believe that addressing the questions above will help us further understand the roles of TETs in the occurrence and development of many diseases.
